# Quantifying The Relationship Between Lockdowns, Mobility, and Effective Reproduction Number (Rt) During The COVID-19 Pandemic in The Greater Toronto Area

**DOI:** 10.21203/rs.3.rs-378425/v1

**Published:** 2021-04-08

**Authors:** Christopher Dainton, Alexander Hay

**Affiliations:** McMaster University; National Institutes of Health

**Keywords:** COVID-19, lockdown, pandemic, mobility

## Abstract

**Background:**

The effectiveness of lockdowns in mitigating the spread of COVID-19 has been the subject of intense debate. Data on the relationship between public health restrictions, mobility, and pandemic growth has so far been conflicting.

**Objective:**

We assessed the relationship between public health restriction tiers, mobility, and COVID-19 spread in five contiguous public health units (PHUs) in the Greater Toronto Area (GTA) in Ontario, Canada.

**Methods:**

Weekly effective reproduction number (R_t_) was calculated based on daily cases in each of the five GTA public health units between March 1, 2020, and March 19, 2021. A global mobility index (GMI) for each PHU was calculated using Google Mobility data. Segmented regressions were used to assess changes in the behaviour of R_t_ over time. We calculated Pearson correlation coefficients between GMI and R_t_ for each PHU and mobility regression coefficients for each mobility variable, accounting for time lag of 0, 7, and 14 days.

**Results:**

In all PHUs except Toronto, the most rapid decline in R_t_ occurred in the first two weeks of the first province-wide lockdown, and this was followed by a slight trend to increased R_t_ as restrictions decreased. This trend reversed in all PHUs between September 6^th^ and October 10^th^ after which R_t_ decreased slightly over time without respect to public health restriction tier. GMI began to increase in the first wave even before restrictions were decreased. This secular trend to increased mobility continued into the summer, driven by increased mobility to recreational spaces. The decline in GMI as restrictions were reintroduced coincides with decreasing mobility to parks after September. During the first wave, the correlation coefficients between global mobility and R_t_ were significant (p<0.01) in all PHUs 14 days after lockdown, indicating moderate to high correlation between decreased mobility and decreased viral reproduction rates, and reflecting that the incubation period brings in a time-lag effect of human mobility on R_t_. In the second wave, this relationship was attenuated, and was only significant in Toronto and Durham at 14 days after lockdown.

**Conclusions:**

The association between mobility and COVID-19 spread was stronger in the first wave than the second wave. Public health restriction tiers did not alter the existing secular trend toward decreasing R_t_ over time.

## Introduction

During the COVID-19 pandemic, public health orders have imposed lengthy restrictions on movement, business operations, and social gatherings. Colloquially referred to as lockdowns, the scope and intensity of such restrictions have been heterogenous and varied widely between jurisdictions worldwide.^1^ Quantitative evaluations of the effectiveness of such measures in attenuating pandemic peaks are rapidly emerging.^2–5^

Many lockdown studies lack a true counterfactual and merely suggest correlation between lockdown measures and temporally associated reductions in case counts.^6^ The use of pre-intervention growth rates to define the success of interventions is similarly limited by the recognition that epidemic curves are time varying and that slowing occurs through natural dynamics even in the absence of intervention.^7^ Likewise, ecological studies suffer from confounding by both known and unknown factors, and ample comparisons exist to favour nearly any hypothesis. Furthermore, it remains unclear how pandemic curves might look based on social distancing recommendations alone rather than legal mandate. As a direct result, there has been intense public debate over whether lockdown policies should be ever more restrictive or rely on voluntary compliance.

Lockdown policies have generated unprecedented controversy as their potential benefits are weighed against direct and indirect harms, as well as impositions on civil liberties.^8^ In particular, extraordinary evidence of efficacy is required to justify restrictions on movement and assembly in liberal democratic societies,^3,9^ and mandatory restrictions may further isolate the marginalized, elderly, and those living alone.

This is particularly relevant in Canada, where the Toronto region has experienced one of the longest periods of business closures in North America, and one of the world’s longest continuous periods of lockdown. There is a need for critical examination of the effects of such policies on viral spread, and the use of colour-coded restriction tiers in the province Ontario offers an unprecedented opportunity for the analysis of the relationship of such restrictions with both movement and viral reproduction rate. Here, we conducted a similar analysis to one performed in Australia^10^ comparing mobility and effective viral reproductive number (R_t_) before and after lockdown measures.

The aim of this observational study was to compare the effect of public health restrictions on mobility and COVID-19 spread in five contiguous public health units within the Greater Toronto Area (GTA), a densely populated urban region within Ontario, Canada.

## Methods

The province of Ontario, Canada is divided into 36 public health units (PHUs) that administer public health services, and of those, Peel (PEL), Toronto (TOR), York (YRK), Halton (HAL), and Durham (DUR) comprise the Greater Toronto Area (GTA), the most densely populated contiguous region in Canada with 6.4 million total inhabitants (548,430 in Halton, 645 862 in Durham, 1,381,744 in Peel, 1,109,909 in York, 2,731,571 in Toronto).^11^

The effective reproduction number (R_t_) is defined as the mean number of secondary cases generated by a typical primary case at a given time tin a population, making it well suited as an indicator of transmission before and after public health interventions.^12^ After November 7, 2020, Ontario imposed a colour-coded tiered approach to the escalation and de-escalation of regional public health restrictions (Table 1), based on weekly incidence, percent positivity, effective reproduction number (R_t_), and outbreak trends. This allowed direct comparison of their relative effectiveness in reducing R_t_.

Official COVID-19 data (daily PCR-confirmed cases) from March 1, 2020 to March 19, 2021 were obtained at the level of the five PHUs from the official websites of each PHU. By March 13, 589 270 Ontarians (4.0%) had at least one dose of vaccine and 285 667 Ontarians (1.9%) had been fully vaccinated^13^, representing approximately 1.85% of the population. Increasingly transmissible variants of concern (VOC) in the GTA ranged from 31.4% in Halton to 49.7% in Durham by the week of March 3–9, 2020.^14^ Weekly estimates of the effective reproduction number (R_t_) at the PHU level were calculated using the EpiEstim R package calculator, found at https://github.com/alechay/covid19-rt. R_t_ was calculated assuming a Poisson distribution, and using a Bayesian framework to estimate credible serial intervals for infections^15^ with the parametric si option in EpiEstim, where the mean and SD of the serial interval were based on previous studies.^16–18^

Google Daily Mobility Reports^19^ are comprised of anonymized and aggregated regional data and use a GPS-linked index of visits and length of stay compared to the pre-pandemic baseline January 3 to March I, 2020. These reports were collected for each PHU for workplaces, residential, parks, grocery and pharmacy, retail and recreation, and transit stations. A global mobility index (GMI) similar to that used in a previous Australian study^10^ was calculated to represent global mobility change, as the mean of each type of mobility *i* in a day *t*:

GMI(t)=∑6i=1Mobilityi/6.

Segmented regressions were used to identify breakpoints in the behavior of R_t_ for each of the PHUs over time (and shifts in COVID-19 transmission trends) using the ‘segmented’ function in R, which employs an algorithm that iteratively fits standard linear regressions to the data and finds points where the properties of the regression (slope, intercept) are significantly changed.^20^ Only regression segments with at least five data points per segment were retained. Intercepts and slopes were calculated for the best model, using separate intercepts at each different segment, allowing for separate identification of increases or decreases in R_t_, and sudden jumps or plunges in daily values.

The median incubation period for COVID-19 is 5.8 days, and 97.5% of patients develop symptoms within 11. 7 days of infection.^21^ Therefore, we selected three scenarios to account for reporting delays from illness onset, testing, and incubation–immediately following policy change, 7 days following policy change, and 14 days following policy change–to relate policy change and mobility change to R_t_.

To evaluate the impact of mobility on R_t_, generalized linear models were estimated using the ‘glm’ function in R for each mobility variable separately. Models with a 0-day, 7-day and 14-day lag of each mobility variable were estimated. We then extracted the mobility regression coefficients for each model.

We then calculated Pearson correlation coefficients between global mobility and R_t_ using the ‘cor.test’ function in R. This allowed us to calculate the association between mobility and COVID-19 spread. All calculations were made using R (version 4.02), with code available on GitHub.

## Results

**Figure 1:**
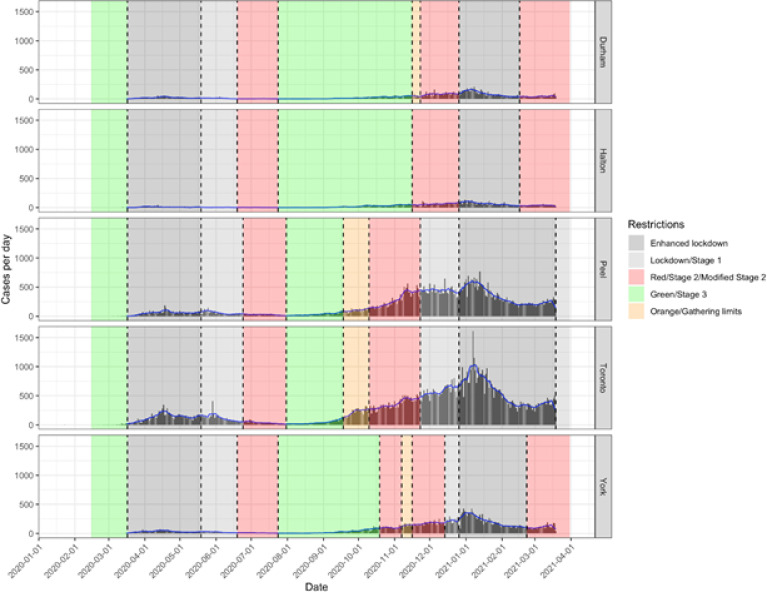
PCR-confirmed daily COVID-19 cases and 7-day moving average in the five public health units in the Greater Toronto Area during the first and second pandemic wave. Shaded areas indicate colour-coded public health restriction tiers.

Figure 1 shows the daily case counts in each PHU in the GTA during the first and second pandemic wave. In general, restrictions were decreased between June 19, 2020 and July 31, 2020, and then progressively increased between September 18, 2020 and December 26, 2020 as daily cases increased in all PHUs during the second wave.

**Figure 2:**
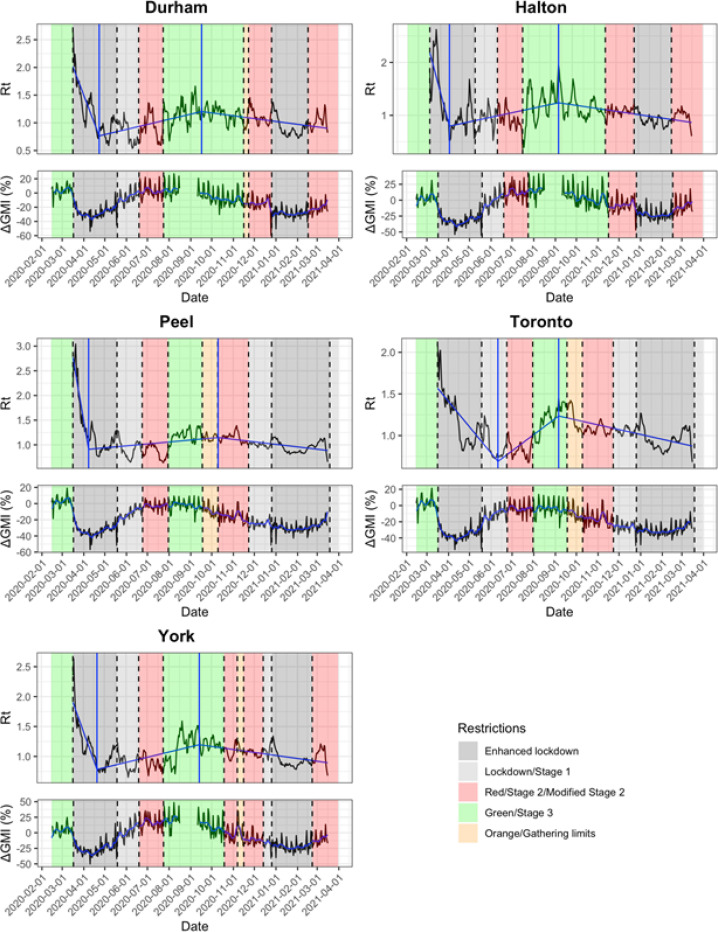
Segmented regressions and effective reproduction number (Rt) for the COVID-19 pandemic in five public health units in the Greater Toronto Area (top graphs). Breakpoints with significant increases and decreases in Rt are indicated as solid blue vertical lines. Global mobility index (GMI) using Google mobility data and 7-day moving average by Greater Toronto Area public health unit (bottom graphs). Shaded areas indicate colour-coded public health restriction tiers.

Figure 2 indicates changes in viral reproduction rate (R_t_) in each PHU based on increasing or decreasing public health restriction tiers. In all PHUs except Toronto, the most rapid decline in R_t_ occurred in the first two weeks of the first province-wide lockdown, and this was followed by a change to increasing R_t_ that began during the lockdown period and continued as restrictions were decreased. This trend changed to decreasing R_t_ in all PHUs between September 6th and October 10th after which there were no further significant slope changes over time. With the exception of Peel, this breakpoint occurred during the lowest level of public health restrictions.

**Figure 3:**
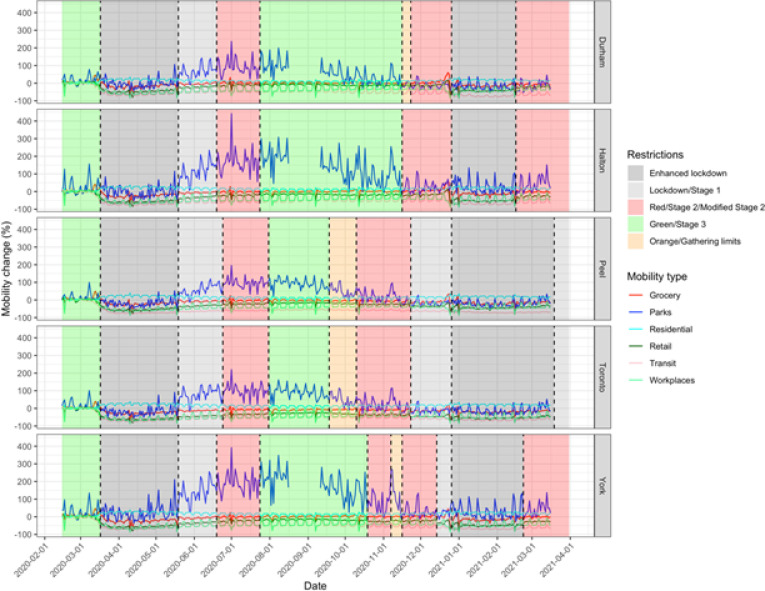
Changes in six types of Google mobility data for five public health units in the Greater Toronto Area. Shaded areas indicate colour-coded public health restriction tiers.

The GMI in all five PHUs decreased with the first lockdown (Fig. 2) and showed weekly cycles with outliers on public holidays. GMI began to increase even before restrictions were decreased. This secular trend to increased mobility continued into the summer, driven by increased mobility to recreational spaces (Fig. 3). The decline in GMI as restrictions were reintroduced coincides with decreasing mobility to parks after September. Mobility in Durham and Halton paradoxically increased in the Red (control) tier, then decreased with enhanced lockdown. Mobility in York decreased prior to reintroduction of restrictions and continued to decrease at the same rate with increasing restrictions. The greatest mobility decreases were seen to retail, transit stations, and workspaces, while mobility to residence increased. Mobility to groceries and pharmacies were largely unchanged throughout the observation period.

**Figure 4:**
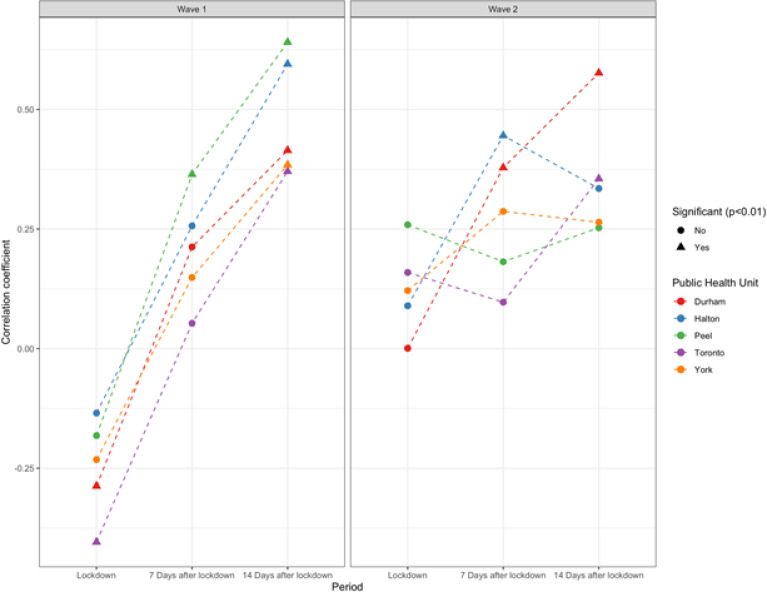
Correlation between global mobility index (GMI) and Rt over three periods of time (right after, 7 days after the lockdown date, and 14 days after the lockdown date) following public health mandated lockdowns in the first and second COVID-19 pandemic wave.

During the first wave, the correlation coefficients between global mobility and R_t_ (Fig. 4) were significant (p < 0.01) in Peel 7 days after lockdown and in all PHUs 14 days after lockdown, indicating a moderate to high correlation between decreased mobility and decreased viral reproduction rates and reflecting that the incubation period brings in a time-lag effect of human mobility on R_t_. In the second wave, this relationship was attenuated, and only significant in Durham and Halton at 7 days after lockdown and in Toronto and Durham at 14 days after lockdown. Paradoxically, in the first wave there were significant correlations between decreased mobility and increased viral reproduction rates in Durham and Toronto in the period immediately after lockdown.

**Figure 5:**
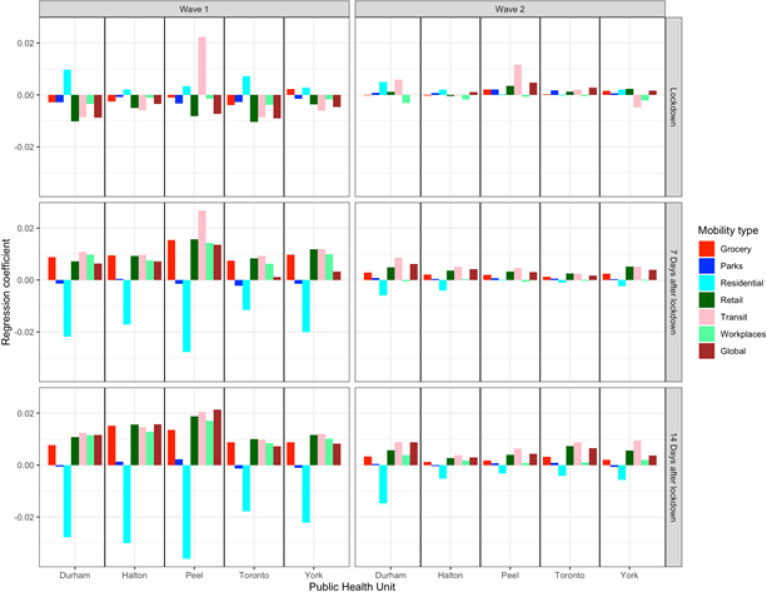
Regression coefficients of each type of mobility in each of five public health units in the Greater Toronto Area over three periods of time (right after, 7 days after the lockdown date, and 14 days after the lockdown date) following public health mandated lockdowns in the first and second COVID-19 pandemic wave.

Figure 5 compares the regression coefficients for each form of mobility in each PHU over three periods of time (immediately after, 7 days after lockdown, and 14 days after lockdown). During the first wave, R_t_ had a negative association with residential mobility at 7 and 14 days after lockdown and a positive association with all other forms of mobility except parks. The associations between R_t_ and mobility were inconsistent in the period immediately after lockdown, reflecting the time lag effect of mobility on spread and the delay of policy intervention. During the second wave, the associations between R_t_ and mobility were inconsistent in all three periods of time, indicating an attenuated relationship.

## Discussion

Using COVID-19 epidemiologic data and Google mobility data, our study relates human mobility, public health restriction policies, and COVID-19 spread in the Greater Toronto Area in Ontario, Canada. Our analysis was similar to that of an Australian study^10^ but its results have important differences. While increased mobility was correlated with increased spread, increased restrictions had inconsistent effects on this mobility. Restrictive measures were associated with a small decrease in virus transmission, but due to the challenges of disentangling multiple confounders, we interpret our findings with caution and link them to the empirical experiences in other countries.

Visual inspection of cases, viral reproductive rate, and mobility level alongside the timeline of policy interventions suggests that the lockdown policies shows little clear indication that these policies altered existing secular trends. This challenges the assumption of a strong association between the current tiered mandatory restrictions and overall virus transmission. With the exception of parks, mobility remained low even after restrictions were lifted, and did not decrease further with increased restrictions. The steep initial decline in mobility even prior to mandatory restrictions closely resembled observations in Sweden, South Korea, Australia, and the United States.^22,23^

Mobility, however, had a 7 to 14-day time lag association with viral spread, which may reflect the viral incubation period, and suggests a dynamic association between mobility and COVID-19 spread. Similar local findings have been reported in New York City^24^, where decreased commuting movements between boroughs as measured by Facebook mobility data were negatively correlated with COVID-19 prevalence. Preliminary data suggests the same is true internationally, where the strong relationship between mobility and virus spread may also be affected by individual preventative behaviours such as social distancing, hygiene, and mask wearing.^25^ Changes in weather conditions could also weaken the association between mobility and virus spread, as people are more likely to spend time outdoors and in parks in summer, where likelihood of transmission is substantially lower.^26^ There were more mixed patterns in mobility-spread correlation after the initial lockdowns, which might reflect diminishing effects due to lockdown fatigue.

### Limitations

Our study has a number of limitations that should influence interpretation. First, Google data uses 3 January 3, 2020 to February 6, 2020 as its baseline, which would bias results if human mobility declined as early reaction to fears of COVID-19 influenced by media reports. Second, it is possible that GMI should be weighted to account for the inherent risk level of each mobility type. Since workspace mobility appears in this study to be higher risk than mobility to parks, for example, it would be reasonable to assign a higher weight to the former. There are also several types of delays to consider that might bias these results: 1) delay between the mobility measure and the date of confirmed cases, 2) the reporting delay from the illness onset date, and 3) delay introduced by incubation and testing. Google data is also a coarse proxy for mobility and social distancing and relies on the movement of those who have a smart device.^22^

The effects of lockdowns may also be confounded by simultaneous media messaging and voluntary changes in behaviour, such as increased mask-wearing. These changes could have caused a temporal autocorrelation of the R_t_ and mobility data, as observations closer in time are likely to be more similar than observations farther apart. Finally, the effects of such lockdowns can be positioned within the context of previous studies indicating a frequently paradoxical effect of more restrictive lockdowns in increasing transmission,^27^-^28^ which may ultimately depend on population density, household density, political climate, travel and border closures, as well as whether sectors of the economy closed by lockdowns are in fact major drivers of spread. The effect of confounders will become increasingly relevant as vaccination campaigns compete with more infectious viral variants during the third wave.

## Conclusion

The association between mobility and COVID-19 spread was stronger in the first wave than the second wave. Public health restriction tiers had no consistent additive effect on altering secular trends to decreasing R_t_. Our findings should be interpreted with caution, since they describe correlation, which may not indicate causal direction between mobility controls and virus spread. Governments should consider the 14-day relationship between mobility and virus spread when reducing restrictions.

## Figures and Tables

**Table 1:** Lockdown restrictions according to the colour-coded five-tier COVID-19 framework implemented in Ontario on November 7, 2020. The provincial restriction tiers used prior to November 7 (stage 1, stage 2, and stage 3) were categorized as grey, red, and green, respectively due to their similarities.

Category	Key restrictions
Green (prevent)/Stage 3	Maximum 10 people indoors, 25 people outdoors for social gatherings Maximum 50 people indoors, 100 people outdoors for organized events
Yellow (protect)	Liquor served only between 9 am and 11 pm Limit of 6 persons seated together
Orange (restrict)	50-person indoor seated capacity limit Limit of 4 persons seated together Liquor served only between 9 am and 9 pm
Red (control)/Stage 2	Maximum 5 people indoors, 25 people outdoors for social gatherings No more than 10 people inside gyms or fitness classes Non-essential retailers operate at 50% capacity Personal care services may operate
Grey (lockdown)/Stage 1	Non-essential retailers operate at 25% capacity Indoor and outdoor dining services prohibited Personal care services closed
Enhanced lockdown	Stay-at-home order Non-essential retailers closed Closure of schools
